# First report of diazotrophic *Brevundimonas* spp. as growth enhancer and root colonizer of potato

**DOI:** 10.1038/s41598-020-69782-6

**Published:** 2020-07-30

**Authors:** Tahir Naqqash, Asma Imran, Sohail Hameed, Muhammad Shahid, Afshan Majeed, Javed Iqbal, Muhammad Kashif Hanif, Shaghef Ejaz, Kauser Abdullah Malik

**Affiliations:** 1 Institute of Molecular Biology and Biotechnology, Bahauddin Zakariya University, Multan, 60800 Pakistan; 2 National Institute for Biotechnology and Genetic Engineering (NIBGE), P.O. Box 577, Jhang Road, Faisalabad, Pakistan; 3 Department of Biosciences, University of Wah, Quaid Avenue, Wah Cantt, Pakistan; 4 Department of Bioinformatics and Biotechnology, Government College University Faisalabad, Faisalabad, 38000 Pakistan; 5 Department of Soil and Environmental Sciences, The University of Poonch, Rawalakot, Azad Jammu and Kashmir Pakistan; 6 Department of Biological Sciences, University of Lahore, Sargodha Campus, 89/2-A ZafarUllah Rd, Shamsheer Town, Sargodha, Punjab 40100 Pakistan; 7 Department of Horticulture, Bahauddin Zakariya University, Multan, 60800 Pakistan; 8 Department of Biological Sciences, Forman Christian College, Ferozepur Road, Lahore, 54600 Pakistan

**Keywords:** Environmental microbiology, Soil microbiology, Biotechnology, Microbiology, Molecular biology

## Abstract

Rhizobacteria contain various plant-beneficial traits and their inoculation can sustainably increase crop yield and productivity. The present study describes the growth-promoting potential of *Brevundimonas* spp. isolated from rhizospheric soil of potato from Sahiwal, Pakistan. Four different putative strains TN37, TN39, TN40, and TN44 were isolated by enrichment on nitrogen-free malate medium and identified as *Brevundimonas* spp. based on their morphology, 16S *rRNA* gene sequence, and phylogenetic analyses. All strains contained *nif*H gene except TN39 and exhibited nitrogen fixation potential through acetylene reduction assay (ARA) except TN40. Among all, the *Brevundimonas *sp. TN37 showed maximum ARA and phosphate solubilization potential but none of them exhibited the ability to produce indole acetic acid. Root colonization studies using transmission electron microscopy and confocal laser scanning microscopy showed that *Brevundimonas *sp. TN37 was resident over the root surface of potato; forming sheets in the grooves in the rhizoplane. TN37, being the best among all was further evaluated in pot experiment using potato cultivar Kuroda in sterilized sand. Results showed that *Brevundimonas *sp. TN37 increased growth parameters and nitrogen uptake as compared to non-inoculated controls. Based on the results obtained in this study, it can be suggested that *Brevundimonas* spp. (especially TN37) possess the potential to improve potato growth and stimulate nitrogen uptake. This study is the first report of *Brevundimonas* spp. as an effective PGPR in potato.

## Introduction

Plant growth-promoting rhizobacteria (PGPR) are a diverse group of microorganisms present in the plant rhizosphere which colonize plant roots. These PGPR facilitate plant growth and significantly improve the yield but constitute only 1–2% of total bacteria present in the rhizosphere^1^. Rhizosphere soil acts as a hot spot for microbial associations because of its nutrient richness and exudates secreted from plant roots, which serve as the food source for rhizobacteria, resulting in the effective recycling of nutrients^2^. Therefore, the isolation, screening, and selection of efficient PGPR are of great interest for their subsequent use as bio-fertilizers for sustaining agro-ecosystems with minimum burden on farmer and environment.

Studies have reported that PGPR from taxonomically diverse genera such as *Rhizobium*, *Pseudomonas*, *Azotobacter*, *Azospirillum*, *Agrobacterium, Klebsiella*, and *Bacillus* have the potential to influence plant growth and yield by using a range of direct and indirect mechanisms. Inoculation with all above-mentioned bacteria have shown a significant positive impact on plant growth and yield both in field and greenhouse conditions^3,4^ around the globe. This steady growth of the global biofertilizers market has reduced the chemical inputs into the environment, thereby, improving the global ecosystem sustainbility^5^. The growth-promoting mechanism of all PGPR are not completely studied yet, however, the processes which are commonly reported to involve in plant growth promotion include (1) nitrogen (N_2_) fixation that convert atmospheric N to plant usable forms (2) phytohormone(s) production which stimulate root proliferation (3) siderophores production that binds and transfers iron to roots of plants (4) solubilization and mineralization of organic and inorganic phosphate and zinc compounds making them available for plant uptake (5) inhibition of phyto-pathogens by producing antibiotics, fungicides, or fungicidal compounds/enzymes (6) and compete with harmful microbes^6,7^. Nitrogen is an essential element required for all plants thus, biological nitrogen fixation is the most important trait of PGPR used for plant inoculation^8^. The bacterial enzyme “nitrogenase complex” is composed of two conserved proteins: one is molybdenum iron protein (di-nitrogenase) encoded by *nif*DK genes and the other iron protein (nitrogenase reductase) encoded by *nif*H gene; catalyzes the reduction of di-nitrogen (N_2_) into ammonium. *Nif*H genes are evolutionarily conserved genes and have gained special importance for phylogenetic studies and identification or detection of diazotrophs (nitrogen-fixing) bacteria in the rhizosphere through cultivation-dependent or independent methods^9^.

Several studies have guaranteed the role of PGPR in enhancing yield and growth of different crops e.g., rice, sunflower, bean, maize and wheat with reduced application of chemical fertilizers and minimal environmental impact^10-14^. Whereas in potato, limited data is available regarding the PGPR-based growth promotion and disease suppression^15-17^. Few studies carried out on potato have reported that PGPR species of genera *Azospirillum*, *Pseudomonas*, *Bacillus*, and *Rhizobium* are involved in nitrogen fixation, phosphorous uptake, indole acetic acid (IAA) production, bio-control activity and induction of systemic resistance against plant pathogens^4,18-21^. The *Brevundimonas* spp. as PGPR has been reported in wheat^22^, but its interaction with potato plant is not known yet.

Potato (*Solanum tuberosum* L.) is the most important food crop worldwide after wheat and rice. The crop demands high fertilizer input (250 kg ha^−1^ nitrogen, 150 kg ha^−1^ phosphorus) to obtain an optimum yield^23^. Such high input of chemical fertilizers produce severe environmental pollution as 50–70% of applied N and P-fertilizers are lost due to run-off, leaching, conversion to unavailable forms and subsequent accumulation in soil or water. It also enhance the cost of crop production as farmers keep on pouring expensive chemicals to the agriculture fields^24^. Alternatively, the biofertilizers based upon PGPR are economically cheap and environmentally safe. The study was based upon the hypothesis that due to the PGP traits especially nitrogen fixation, *Brevundimonas* spp. obtained from potato rhizosphere would improve potato growth. The study involves the isolation, screening, identification and characterization of *Brevundimonas* spp. from two different sites of Sahiwal, Pakistan and contribution to increase plant growth. This is the first report revealing the root association of *Brevundimonas* spp. with potato along with its plant growth promoting effects.

## Results

### Soil analysis

The two samples of soil collected from the Sahiwal (Sw) region showed similar loamy soil texture with a slight change in pH and total percent nitrogen. Electrical conductance (EC) of soil ranged from 0.6 to 1 with approximately two fold difference. The percentage of organic matter, total mineral N and total mineral P in Sw2 soil sample were substantially greater than Sw1. However, Sw1 soil showed higher levels of phosphorous and potassium than Sw2 soil. All the measured soil physico-chemical properties are given in Table 1.

**Table 1:** Physico-chemical analysis of Sahiwal soil samples from where the bacteria were isolated.

Physicochemical properties of soil	Sw1	Sw2
EC (d S m^−1^)	1	0.6
Soil texture	Loam	Loam
Soil pH	8.3	8.4
Organic matter (%)	1.04	1.53
Total N (%)	0.07	0.06
Total mineral N (mg kg^−1^)	4.13	5.31
Total P	50.6	29.3
Total mineral P (mg kg^−1^)	220	140
Total K	36	30

Sw1, Soil sample 1; Sw2, soil sample 2.

### Morphological characteristics of bacteria and 16S *rRNA* based identification

Four bacterial strains were obtained on semi-solid NFM medium from enrichment cultures of soil samples based on their shape, size, colony and color. All the isolates were Gram-negative, medium-sized short rods and showed round shaped colonies expect TN44, which showed irregularly shaped colonies. The isolates showed variable colony colors on LB. Isolate TN37 formed pale yellow, TN39 formed creamy, TN40 formed white while TN44 formed grayish colored colonies. The Table 2 summarizes the details of cell morphology, colony morphology and gram’s reaction of these bacterial strains. In PM2A, BIOLOG microplate assay, only 9 of 95 different carbon sources were utilized by bacterial isolateTN37 showing its limited metabolic potential associated with potato roots (Supplementary Table S1).

**Table 2:** Morphological characteristics and identification of bacterial isolates from rhizosphere of potato, based on 16S *rRNA* gene sequence analysis.

Isolate code	Isolation site	Colony morphology	Cell morphology	Gram’s reaction	ARA (nmoles mg^−1^ protein h^−1^)	Closest GenBank match of 16S *rRNA* with %	GenBank accession number
TN37	Sahiwal	Medium, round, pale yellow	Short rods	−ve	135.07 ± 8.97	*Brevundimonas naejangsanensis* strain HWG-A15(99%)	LN833470
TN39	Sahiwal	Medium, round, creamy	Short rods	−ve	127.56 ± 11.63	*Brevundimonas terrae* strain KSL-145 (99%)	LN833471
TN40	Sahiwal	Medium, round, white	Short rods	−ve	0	*Brevundimonas *sp. X60 (99%)	LN833472
TN44	Sahiwal	Medium, irregular, grayish	Short rods	−ve	93.36 ± 6.38	*Brevundimonas *sp. MM68May (99%)	LN833475

Values are means of three independent replicates ± SE.

ARA, acetylene reduction assay.

**Figure 1:**
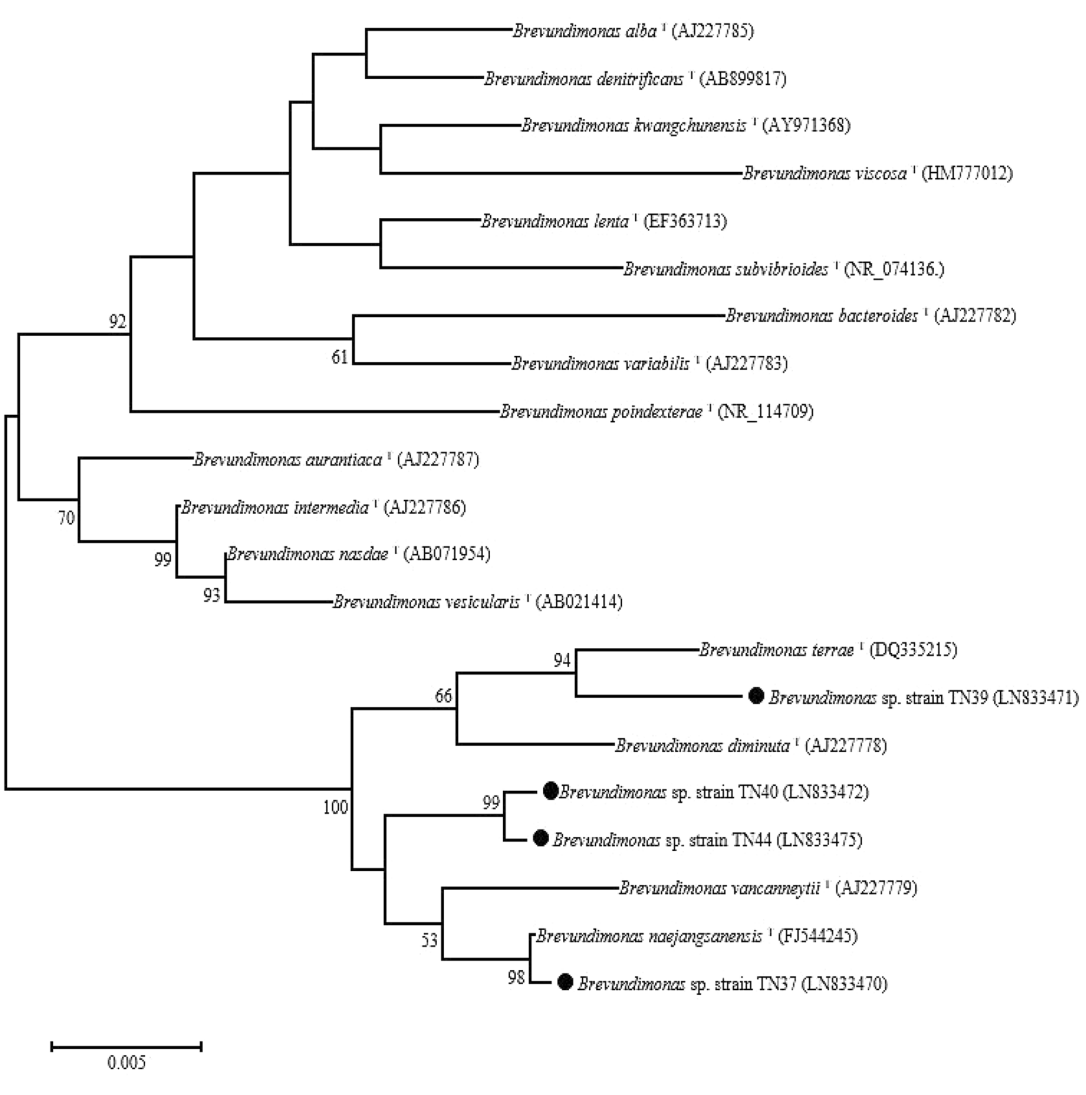
Phylogenetic tree based on 16S *rRNA* sequences (1165 bp) of *Brevundimonas* spp. isolated from potato rhizosphere (filled circle) and published sequences. Numbers present at the branching points are bootstrap values > 70%. Bar represents sequence divergence of 0.005 nucleotides.

Based on 16S *rRNA* sequence analysis, the bacterial isolates were identified as *Brevundimonas* spp., showing 99% similarity with gene sequences of different species of the genus *Brevundimonas* in the Genbank (Table 2). The sequences of 16S *rRNA* have been submitted in the nucleotide database of Genbank under the accession numbers LN833470, LN833471, LN833472 and LN833475. For phylogenetic analysis, neighbor-joining method was adopted with bootstrap values greater than 50 (Fig. 1). Isolate TN37 was clustered with *B. naejangsanensis* ^T^(FJ544245), TN39 clustered with *B. terrae* ^T^(DQ335215) while isolate TN40 and TN44 were branched with *B. vancanneytii* ^T^(AJ227779) and *B. naejangsanensis* ^T^(FJ544245) (Fig. 1).

### *Nif*H gene analysis and nitrogenase activity

**Figure 2:**
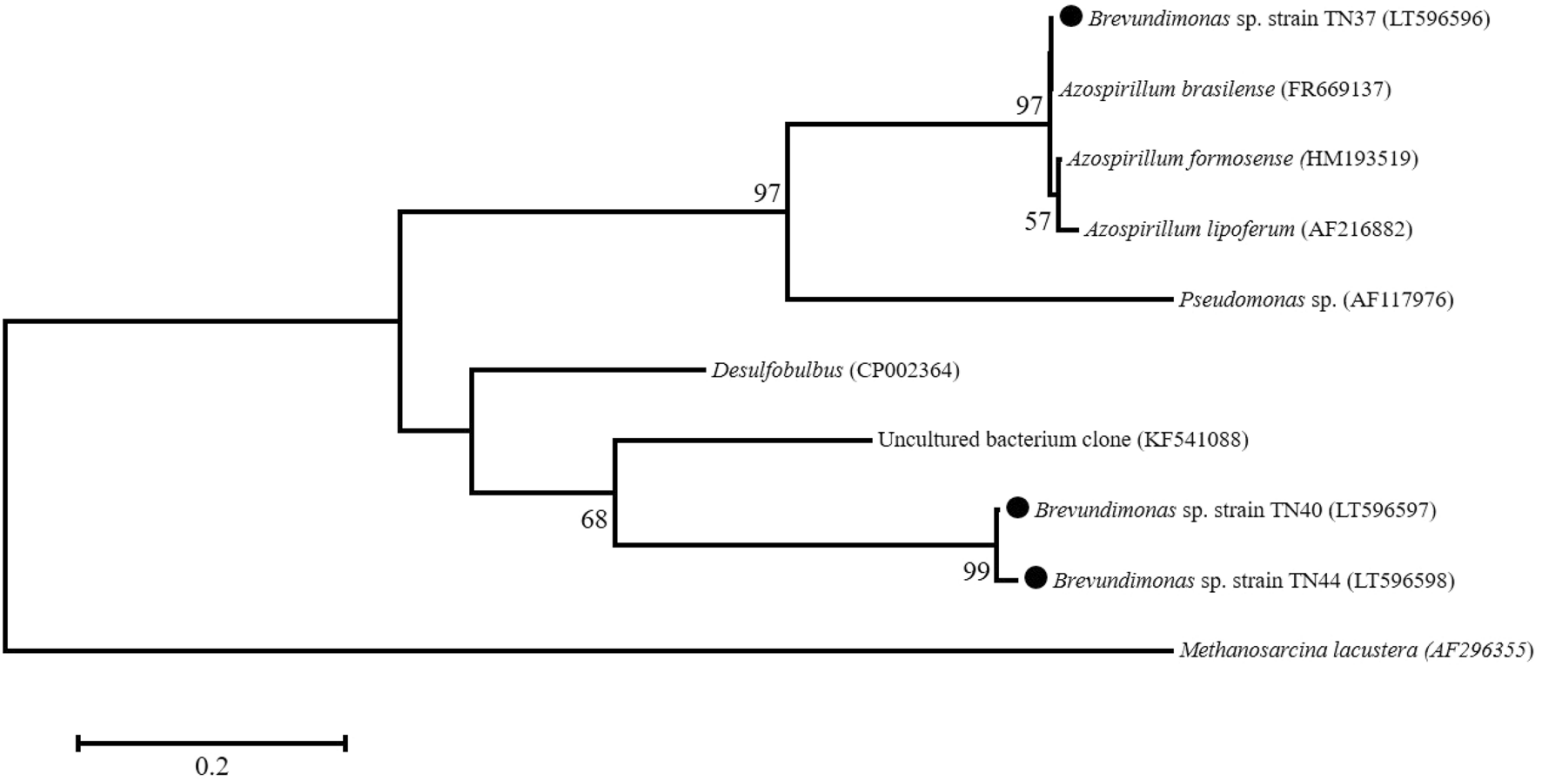
Phylogenetic tree based on *nif*H gene sequences (300 bp) of *Brevundimonas* spp. strains isolated from potato rhizosphere (filled circle) and obtained from published sequences. Numbers present at the branching points are bootstrap values > 90%. *Methanosarcina lacustera* (AF296355) was kept as root. Bar represents sequence divergence of 0.2 nucleotides.

For evaluating nitrogen-fixing potential of these isolates, genomic DNA was extracted and the *nif*H gene was amplified. Of four, three isolates TN37, TN40 and TN44 showed amplification of *nif*H gene with a product size of 300 bp while no amplification of *nif*H gene was observed in TN39 isolate. The 300 bp fragment was sequenced and obtained sequences were compared with already submitted sequences in the GenBank database. The BLAST and phylogenetic analyses showed that *nif*H gene sequence amplified from *Brevundimonas* sp. strain TN37 was highly similar to *nif*H of *A. brasilense* Gr42 (Acc. No. FR669137) as it made cluster with *Azospirillum* sp. and subcluster with *A. brasilense*. However, *Brevundimonas* spp. strains TN40 and TN44 showed 99% similarity to the *nif*H gene sequence of uncultured bacterium clone OTU-31 (Acc. No. KF541088) (Table 3). Though, both strains showed similarity to the same *nif*H gene in data bank but in phylogenetic analysis they made subcluster at 99% with each other (Fig. 2). The *nif*H genes of *Brevundimonas* spp. strains TN37, TN40 and TN44 were submitted to the database under accession numbers: LT596596, LT596597 and LT596598, respectively. The *nif*H gene presence shows that these isolates may have the potential of nitrogen fixation which was later validated by in vitro assay. The isolate TN37, TN39 and TN44 showed nitrogenase activity measured by ARA. Isolate *Brevundimonas* sp. TN37 showed the highest activity (135 nmoles mg^−1^ protein h^−1^) while we could not detect any ARA activity by *Brevundimonas* sp. strain TN40 (Table 2).

**Table 3:** *Nif*H gene sequence similarity of nitrogen-fixing bacteria from rhizosphere of potato.

Strain	Closest GenBank match based on *nif*H sequence (% identity)	Accession nos.
*Brevundimonas* sp. TN37	*Azospirillum brasilense* Gr42 (100)	LT596596
*Brevundimonas* sp. TN40	Uncultured bacterium clone OTU-31 (83)	LT596597
*Brevundimonas* sp. TN44	Uncultured bacterium clone OTU-31 (83)	LT596598

### IAA production and phosphate solubilization

**Figure 3:**
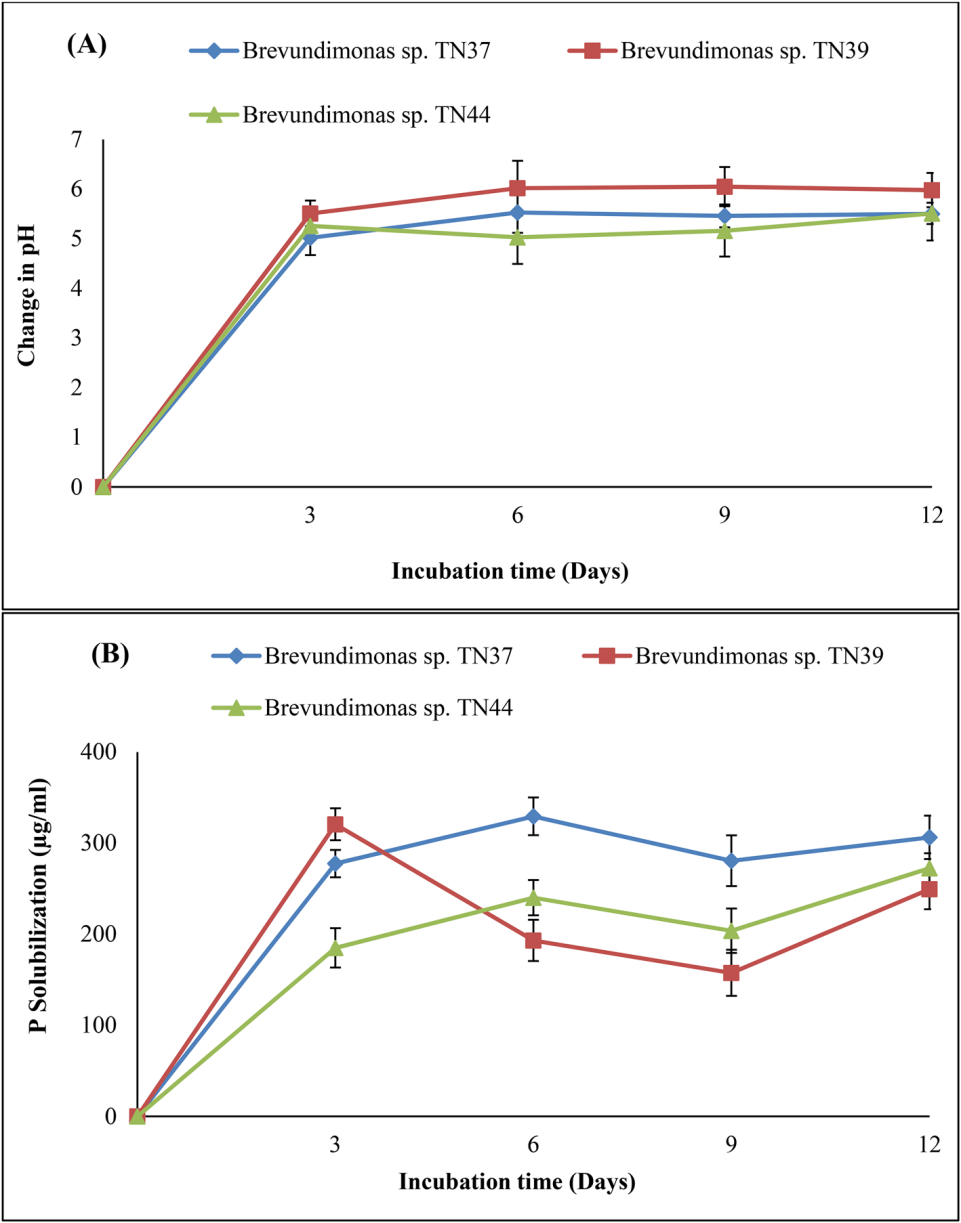
Relationship between (**A**) pH change and (**B**) phosphate solubilization of *Brevundimonas* spp. strains TN37, TN39 and TN44 in Pikovskaya’s media with time. Values are mean of three biological replicates. The standard deviations of mean values are represented as bars.

The results of Salkowski assay showed that bacterial strains were unable to produce IAA either in the absence or presence of l-tryptophan. All bacterial strains except TN40 showed the ability to solubilize phosphorous in the growth media. Out of which strain TN37 showed the maximum P-solubilization potential and solubilized 306.25 µg mL^−1^ of phosphorous while TN39 and TN44 solubilized 249.37 µg mL^−1^ and 272.50 µg mL^−1^ of phosphorous in the growth medium after 12 days with the consequent decrease in pH up to 5.5, 5.98 and 5.51, respectively (Fig. 3A,B).

### Rhizosphere survival, root colonization and plant growth-promoting potential of *Brevundimonas* sp. TN37

Being the best among four bacteria, rhizosphere survival, root colonization and plant inoculation potential of *Brevundimonas *sp. TN37 was checked in vitro and in vivo. The bacterial population of strain TN37 changed dynamically in potato rhizosphere at different stages of plant growth. At day zero (1st day of inoculation), the colony forming units of *Brevundimonas* sp. TN37 was 7.57, which decreased gradually upto 5.56 CFU on the final harvesting (Day 60 of inoculation) (Fig. 6E). In uninoculated control plants, no bacterial cells were observed in their rhizospheric region.

**Figure 4:**
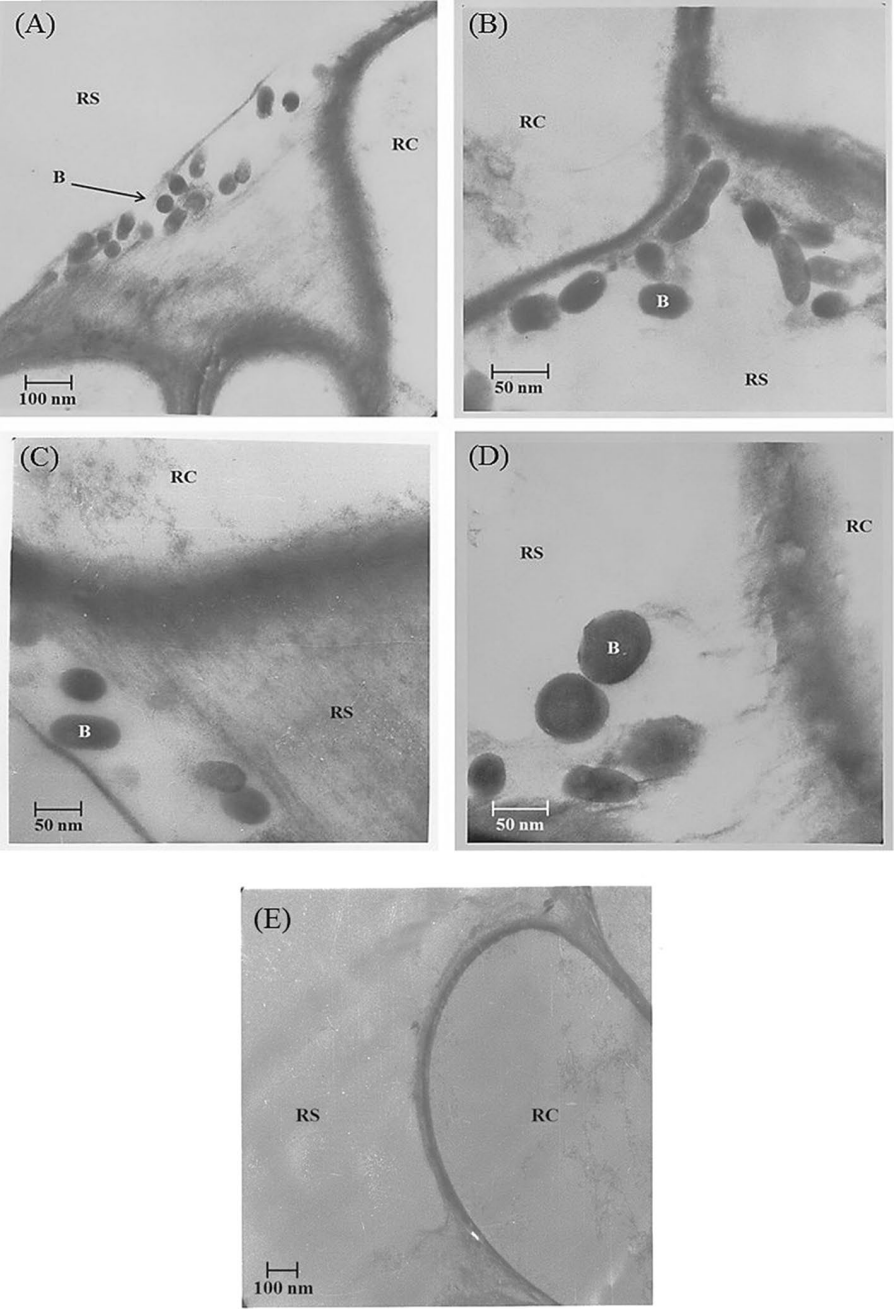
Electron micrographs of ultra-thin sections of potato root inoculated with *Brevundimonas* sp. TN37, 30 days post inoculation. *RS* Rhizosphere, *RC* root cell and *B* Bacterium. *Brevundimonas* sp. TN37 colonized over the root surface of potato. Bacterial cells form sheets in the grooves, formed by the root cells (**A**,**B**) and also closely attached to the cell wall of plant cells (**C**,**D**) and in control uninoculated plants no bacterial cells were observed (**E**).

**Figure 5:**
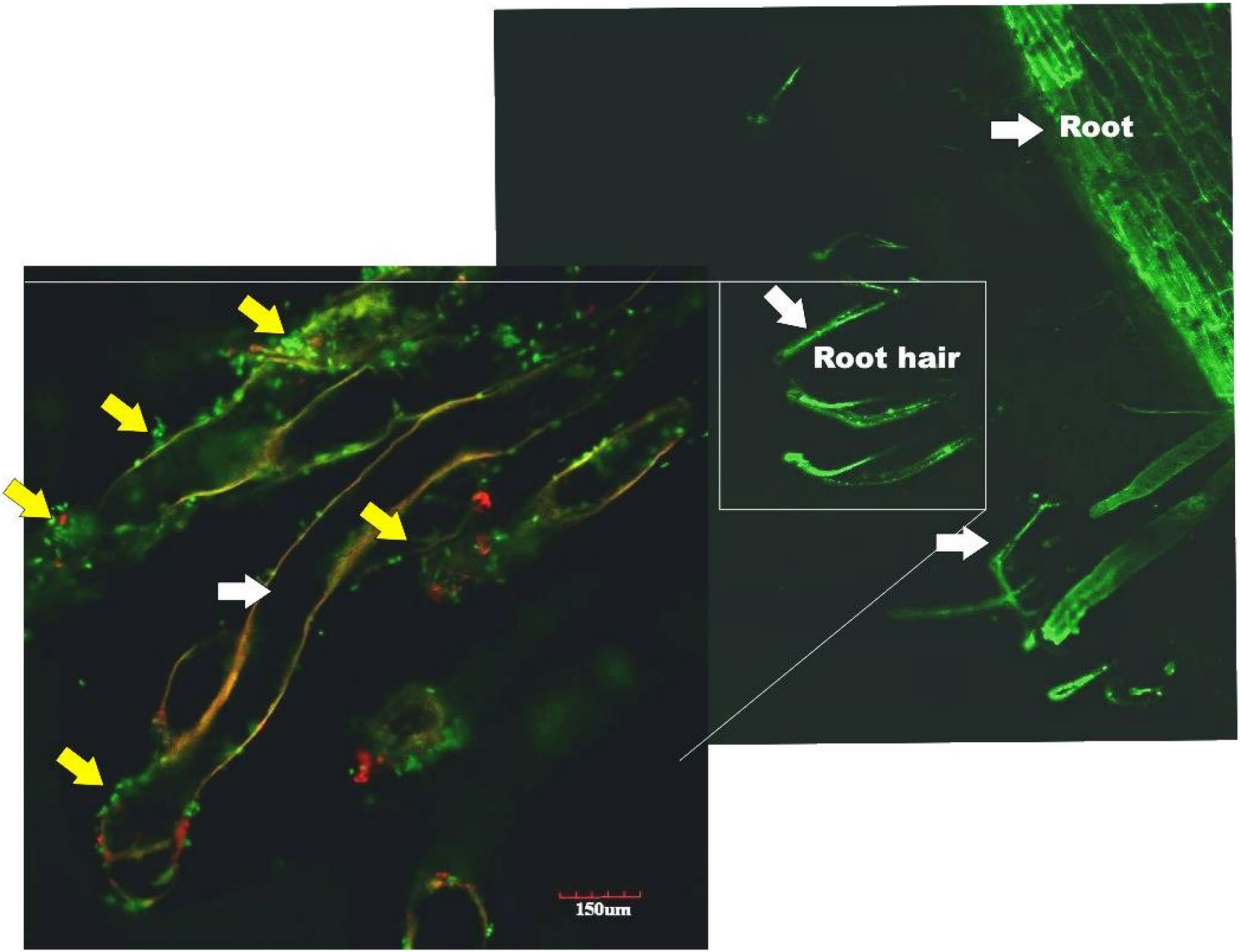
Confocal image of potato (variety Kuroda) root inoculated with YFP-labelled *Brevundimonas* sp. TN37, in sterile sand 25 days post inoculation. Primary root tips and root hairs of potato were preferred targets of colonization by *Brevundimonas* sp. TN37. Macro-colonies, forming bacterial aggregates were observed on the epidermal root cells that endorse the association of strain TN37 with potato.

Electron microscopic analysis confirmed the CFU data. Observation of ultrathin sections of potato root after 30 days post-inoculation under TEM showed that *Brevundimonas *sp. TN37 was an inhabitant of the root surface (i.e., rhizoplane) (Fig. 4). The bacterium formed sheets in the grooves, formed by the root cells and was closely attached to the cell wall of plant (Fig. 5A–D). No bacterial cells were observed in or on the root surface or in rhizosphere of uninoculated control plants (Fig. 5E). Confocal microscopic analysis further validated the root colonization potential of *Brevundimonas* sp. TN37. The potato root inoculated with YFP-labelled *Brevundimonas* sp. TN37, in sand culture 25 days post-inoculation showed that primary root tips and root hairs of potato were preferred sites of colonization by *Brevundimonas *sp. TN37. Macro-colonies of bacterial aggregates were observed on the root epidermal cells. The microscopic observations support the root colonization potential and rhizosphere competence of *Brevundimonas* sp. strain TN37 in potato (Fig. 5).

**Figure 6:**
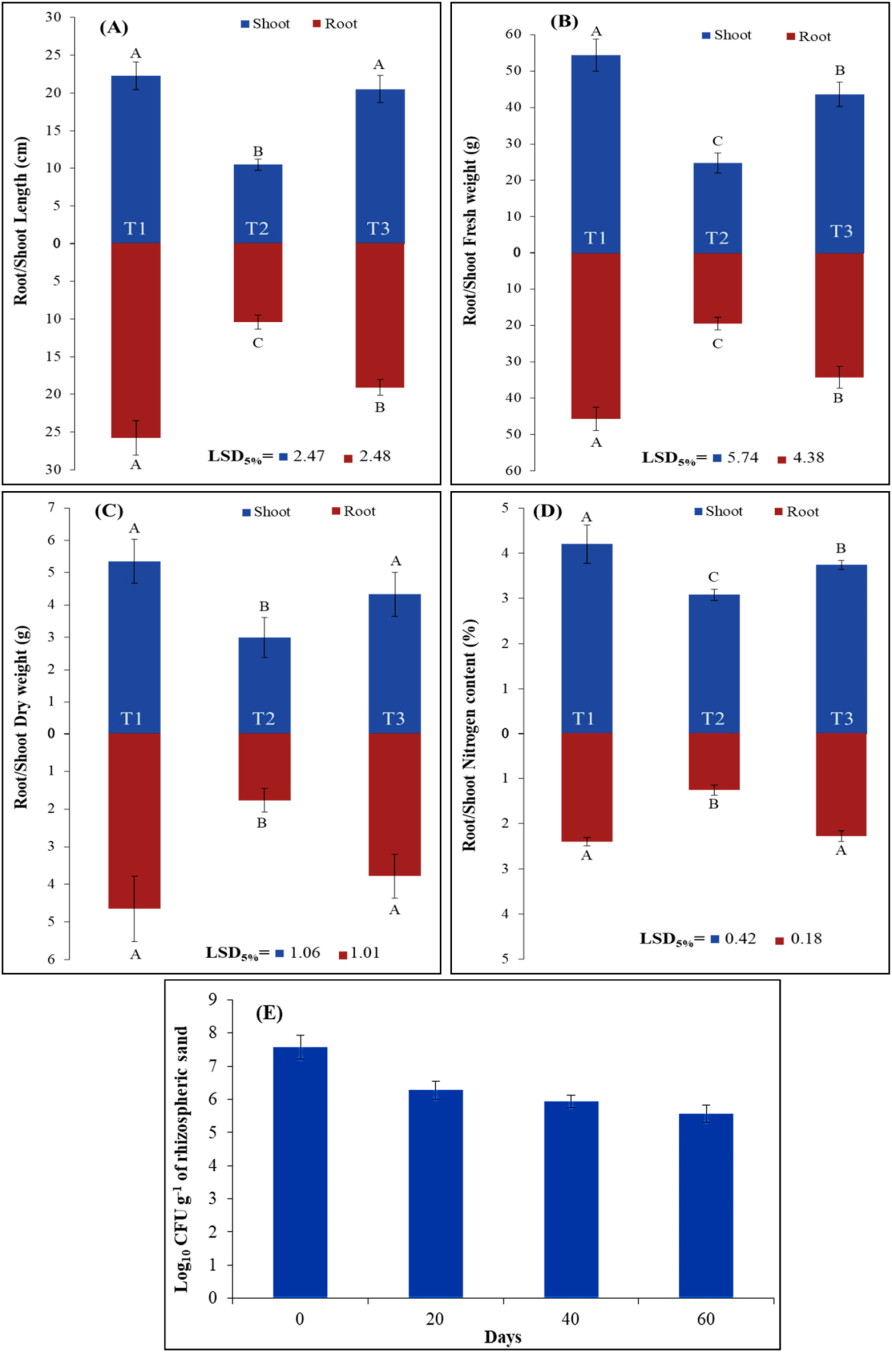
Inoculation response of *Brevundimonas* sp. TN37 on shoot and root: (**A**) fresh weight, (**B**) dry weight, (**C**) length, (**D**) nitrogen content, and (**E**) population dynamics and survival of *Brevundimonas* sp. strain TN37 inoculated to potato rhizosphere. T1 = positive control (recommended full dose of N^F^); T2 = negative control (N^0^ = without nitrogen); T3 = inoculated treatment with bacterial isolate TN37 + N^0^; LSD = Least Significant Difference; Letters A, B and C show significant difference between means of different treatments. Values are mean of three biological replicates. The standard deviations of mean values are represented as bars.

The potential of *Brevundimonas* sp. TN37 isolate in promoting plant growth was evaluated by inoculating potato plants in pots*.* The data showed a positive effect on biomass and nitrogen content of potato plant when inoculated with *Brevundimonas* sp. TN37. The effect of *Brevundimonas *sp. TN37 inoculation on shoot and root fresh weight, total nitrogen contents of shoot, and root length were significantly higher than negative control (Fig. 6A–D). This increase over non-inoculated (negative) control can be attributed to biological nitrogen fixation potential of the strain.

## Discussion

Nitrogen is one of the most important nutrients for potato growth because both the deficiency and the excess of N may hamper the crop cycle and yield^25^. Excessive N-fertilization results in deterioration of ecosystem and global environment. The beneficial bacteria residing in the rhizosphere can minimize the fertilizer inputs in to agriculture along with reduced cost of crop production and environmental pollution. Present study describes the potential of a nitrogen-fixing potato plant-beneficial bacterium *Brevundimonas* sp. TN37 for improving growth and nitrogen uptake under limited-nitrogen fertilizer.

The soil sample from two different locations of Sahiwal which is a major potato growing region showed higher organic matter, nitrogen, phosphorous and pH (Table 1). Higher nitrogen and phosphorous contents shows that the soils receive excessive chemical fertilizers which ultimately degrade the soil physical and biological functioning. Characteristics of soil directly influence microbial diversity, plant–microbe interaction, roots development and plant growth^26-28^. Four different *Brevundimonas* spp. strains were isolated on NFM and further confirmed by phylogenetic analysis.

An important property of rhizobacteria is their nitrogen-fixing ability which directly improves plant growth because N is an essential macronutrient required for plant growth and development. In the present study, isolated strains *Brevundimonas* spp. TN37, TN39 and TN44 showed ARA activity, suggesting their ability to fix nitrogen. Furthermore, these strains showed the presence of *nif*H gene in their genomes. *Brevundimonas* sp. TN39 strain, although, showed ARA activity but did not show amplification of *nif*H gene with the reported universal primers. It is already reported that these universal primers do not amplify *nif*H gene in several strains of diazotrophs^29^. Similar discrepancies have been reported for ARA results and *nif*H gene profiles^30,31^. To get more clarity in such cases, genome sequencing can help to elucidate the disparity. The amplification of *nif*H gene in other three *Brevundimonas* spp. shows that the strains contain the gene and have potential for nitrogen fixation. The sequence-similarity of *nif*H gene of *Brevundimonas* sp. TN37 with *Azospirillum* sp. shows that the gene may be acquired from that genus through horizontal gene transfer or from a common ancestor during evolution. Higher ARA activity of *Brevundimonas* sp. strain TN37 might be due to the sequence similarity with *Azospirillum* because this genus is a well-known and widely used bacterial diazotroph^32^. On the other hand, the *nif*H genes of the other two *Brevundimonas* spp. TN40 and TN44 may represent novel genes as they do not have any cultured homologs in the database. *Nif*H is evolutionarily conserved and could also be used for the identification of nitrogen-fixing rhizobacteria^9^ but it cannot be used for specie delineation. ARA is a widely accepted test for evaluating the nitrogen-fixing potential and nitrogenase activity^33^ but in present study, *Brevundimonas* sp. TN40 did not show any ARA activity although the strain showed the amplification of *nif*H gene. The reason for this may be the *invitro* conditions which might not be suitable for this strain to perform its nitrogen fixing activity. Study also reported that nitrogenase activity is affected due to culture medium, growth stages and conditions or possibly in some strains the activity is active only in planta^34^. Different studies reported the transfer of nitrogen between roots of different crops and nitrogen-fixing bacteria^35-37^.

The evaluation of other plant growth promoting traits revealed that all strains except TN40 were capable of solubilizing phosphorous (tri-calcium phosphate) of which *Brevundimonas* sp. TN37 showed the maximum potential of P-solubilization (306.25 µg mL^−1^) within 12 days. Plant growth promoting rhizobacteria have the potential of P-solubilization convert the inaccessible soil P into plant-available P-form that are taken up as essential nutrient source for plant growth and development^38^. The bacteria did not show the ability to produce IAA. Although IAA-production ability is widespread among rhizobacteria but its production depends on several factors e.g., the potential of isolates to utilize tryptophan, sampling location, pH, oxygen availability, carbon conditions^39^, and the incubation time^40^. Longer incubation time causes decrease in nutrients found in growth media and possibly the amount of IAA produced consumed again by bacterial cells for their own growth. Thus, it can be suggested that may be due to one of these reasons isolates were unable to produce IAA.

Rhizosphere competence and root colonization is an important feature of any candidate strain for biofertilizer production. *Brevundimonas* sp. TN37 was found to be a root-colonizer of potato forming strong root- associations. The bacterial cell density was high in the rhizosphere, where root hairs were preferred sites for the early colonization. Root hairs are identified as sites of increased rhizodeposition and are supposed to be involved in specific attachment and eliciting chemotaxis response^40-42^. Micro and macro colonies were also observed in the junctions of primary and secondary roots which may be because it provides a better niche, support for attachment and allow nitrogen fixation to take place. Cells of *Brevundimonas* sp. TN37 were distributed all over the root zone which is probably due to variation in the root exudates at different places within the root system^43^.

Being a P-solubilizing diazotroph and root colonizer *Brevundimonas* sp. TN37 was evaluated for its growth-promoting potential in potato. The increase in biomass and high nitrogen contents due to the inoculation of *Brevundimonas* sp. TN37 can be attributed to diazotrophic ability of this bacterium. The study conducted in canola^3^ plant showed increased biomass when inoculated with different bacterial strains as *Achromobacter*, *Chryseobacterium*, *Pantoea*, *Pseudomonas* and *Klebsiella.* Similar findings were reported in wheat and Bt-cotton when inoculated with PGPR^22,44^. Thus, it can be suggested that nitrogen plays an important role in growth promotion because its deficiency influences the yield and growth of crops. This study is the first report of *Brevundimonas* spp. as PGPR in potato crop.

## Conclusion

This study demonstrates the isolation, screening and molecular identification of *Brevundimonas *spp. and its role in growth promotion. Potato requires high inputs of N and P fertilizers; therefore, the presence of such P-solubilizing, nitrogen-fixing bacterium will help to increase its biomass by making nitrogen and phosphorus available from the atmosphere or rhizosphere. Our findings imply that *Brevundimonas* sp. TN37 has the potential of nitrogen fixation as well as P-solubilization that helps to improve plant growth and can maintain soil fertility.

## Materials and methods

Soil samples from the rhizosphere of potato plants were collected in plastic bags (25 × 30 cm) from two different sites of Sahiwal, Punjab, Pakistan. Samples were kept on dry ice for further experimentation and transferred to the laboratory. All the experiments were repeated three times for validation of results. The bacterial population of rhizospheric soil was determined by the basic technique of serial dilution^45^. For isolation of nitrogen-fixing bacteria from the rhizosphere, soil with roots (0.1 g) was added to 1.5 mL Eppendorf tubes containing NFM semi-solid medium^46^ and were incubated at 28 ± 2 °C for 48 h. Culture from the semi-solid medium was then streaked on NFM agar plates and LB agar plates. Single and purified colonies having different morphological characters were obtained and grown for 24 h at 28 ± 2 °C on respective media and were preserved in glycerol (20%) at − 80 °C.

### Physio-chemical analysis of soil samples

The physio-chemical properties of soil samples were evaluated after drying at 40 °C for 24 h followed by sieving through 2 mm mesh. For determination of soil texture, soil sample (50 g) was soaked overnight in 40 mL of sodium hexa-metaphosphate solution (1%) with 150 mL of distilled H_2_O (dH_2_O). Bouyoucous Hydrometer was used for recording preliminary reading after 40 s of stirring while final reading was obtained after 2 h. The international textural classification system was used to assign class to soil texture^47^. Soil paste was prepared for measuring pH. 250 g of soil and dH_2_O was allowed to stand for 1 h. Then the pH of this paste was measured by pH meter (JENCO Model-671P)^48^. The electrical conductivity of soil was recorded from the clear extract using EC meter (Jenway). The extract was obtained from soil paste using a vacuum pump using previously described protocol of Rhoades et al.^49^. Organic matter of soil was measured by making soil solution from 1 g soil sample, 1 N potassium dichromate (10 mL) solution, conc. H_2_SO_4_ (20 mL), dH_2_O (150 mL) and 0.5 N FeSO_4_ solutions (25 mL). This solution was then titrated with potassium permanganate solutions (0.1 N) which gives pink color as an endpoint^50^. To measure total soil nitrogen (N), 10 g of soil was digested with conc. H_2_SO_4_ (30 mL) and 10 g of digestion mixture (CuSO_4_:FeSO_4_:K_2_SO_4_ = 0.5:1:10) in Kjeldahl’s digestion tubes ^51^. This digested material was then cooled and an aliquot of 10 mL was used for distillation of ammonia from this, with boric acid solution (4%) and methyl red and boromocresol green as indicators in a receiver. In the distillation flask, NaOH solution was added to increase the pH of the contents. Gunning and Hibbard’s method as used for titration of the material using N/10 H_2_SO_4_. Titration was done after distillation in the receiver with the micro Kjeldahl apparatus^51^. Extractable phosphorus was measured by the method described by Olsen using a spectrophotometer^52^. To determine the extractable K, soil solution (5 g) was prepared with ammonium acetate solution (1 N) and the volume was made up to 100 mL. After continuous shaking, the extractable K was measured on Flame Photometer from extracts, obtained from filtering suspension by Whatman filter paper No. 1^53^.

### Morphological characterization

For colony morphology, bacterial isolates were streaked onto LB agar plates followed by 24 h incubation at 28 ± 2 °C. Light microscope (Nikon LABOPHOTO-2, Japan) was used to examine the shape and motility of bacterial isolates. For Gram staining, method described by Vincent was followed^54^.

### Biochemical characterization

*Azospirillum brasilense* strain ER20 (Accession no. HE662867)^55^ was obtained from the NBRC culture collection NIBGE, Faisalabad, Pakistan and was used as a reference strain or a positive control for in vitro analysis. Nitrogen-fixing ability of bacterial strains was evaluated using ARA (Acetylene Reduction Assay)^56^. Each isolated strain was grown for 72 h in NFM semi-solid medium at 28 ± 2 °C and their nitrogenase activity was evaluated by gas chromatograph fitted with flame ionization detector and Porapak N column^57^. Activity was measured as nmoles of ethylene produced per milligram per hour of the protein. The method described by Bradford^58^ was adopted for the estimation of protein concentration.

IAA production was measured by calorimetric method. The test was conducted in the presence or absence of IAA precursor (l-tryptophan). Purified single colonies of bacterial isolates were added in Erlenmeyer flasks (250 mL) containing LB broth (100 mL) supplemented with 100 mg L^−1^ of l-tryptophan. The culture was incubated for 48 h at 28 ± 2 °C with continuous shaking, following 15 min centrifugation at 4,000×*g*. The collected supernatant was filtered using nylon filters (0.2 µm) and this filtered supernatant (100 µL) was mixed with Salkowski reagent (100 mL) following 20 min incubation at room temperature. The IAA production was quantified using a standard curve against known concentration of IAA at λ = 540 nm^59^. The quantitative analysis of phosphate solubilization of isolated strains was done by inoculating them in Pikovskaya’s broth (100 mL) in triplicate following incubation at 28 ± 2 °C in the orbital shaker for 288 h (12 days) at 150 rpm. 20 µL of bacterial culture was harvested from each flask at 72, 168, 240 and 288 h post inoculation, following 10 min centrifugation at 13,000×*g*. The phosphate solubilizing activity of supernatant was determined by phospho-molybdate blue color method with the help of a spectrophotometer at λ = 882 nm^60^.

### Molecular characterization

#### Sequence analysis of 16S *rRNA *and *Nif*H genes

DNA of the pure culture was isolated using microbial DNA isolation kit (MoBio, CA). Following the manufacturer protocol, genomic DNA was obtained and stored at − 20 °C. The amplification of 16S *rRNA *of bacterial isolates was done by PCR using the primers 968F^61^ and 1406R^62^ ensuring the conditions described by Shahid et al.^11^. A nested PCR was conducted for the amplification of *nif*H gene from the pure cultures^63^ using primer set FGPH19^64^ and PolR^65^. The 2nd PCR was done by using primers set of PolF and AQER and product of 1st PCR as the template^65^. The products of bacterial isolates and *nif*H gene were analyzed on ethidium bromide stained agarose TAE gel with 1 kb ladder (Fermentas, Germany). Following the manufacturer's instructions, PCR products were purified using PCR Clean-up System (Promega) and Wizard SV Gel and sent for sequencing to LGC Genomics, Berlin. The sequenced products of *nif*H gene and 16S *rRNA* gene sequences were analyzed using a sequence scanner software package.

#### Phylogenetic analysis

For phylogenetic analysis, MEGA6 software package was used. The sequences of isolated strains were analyzed and compared by alignment tool CLUSTAL W, using the downloaded closely related sequences from the NCBI database^66^. Phylogenetic analysis was performed using neighbor-joining method, but the bootstrap values of 70% or greater were retained for representing well-supported nodes^67^.

### Phenotypic microarrays

BIOLOG micro-plates were used to evaluate the metabolic potential of bacterial isolates^68^. Strains were grown for 48 h on LB agar plates at 28 ± 2 °C. Culture was collected in Eppendorf tubes containing DEPC water (1 mL) and starved for 3 h. After that, culture was mixed with inoculation fluid IF-0 and redox indicator according to the manufacturer’s instruction. In 96 wells containing carbon utilization plate (PM2A), 100 µL of culture was added following incubation for 24 h at 28 ± 2 °C and were detected on VERSA max micro-plate reader (Molecular Devices, USA) having softmax pro-software for both qualitative and quantitative evaluation^69^.

### Rhizosphere competence and root colonization studies using TEM

Bacterial population in potato rhizosphere at different time intervals was recorded by serial dilution plating^70^. Roots of these plants were rinsed with sterilized water and cut into approximately 1–3 cm pieces which were embedded in water agar (1.5% w/v) in the form of 2–3 mm^3^ cubes and were placed in the 2 mL Eppendorf containing glutaraldehyde (5%) as fixative. After 16–18 h, glutaraldehyde was replaced with 0.2 M of PIPES buffer [3 g PIPES, 0.58 g NaCl, 1 M NaOH, 0.2 g MgCl_2_.6H_2_O, pH 6.8] at pH 8.0. Samples were prepared following the protocol described by Naqqash et al.^4^ and were examined under a transmission electron microscope (TEM; JEOL JEM1010, USA).

### Transformation of bacterial isolates with yellow fluorescence protein (YFP)

Electro-competent cells of selected bacterial isolates were prepared for studying root colonization using Confocal Laser Scanning Microscope (CLSM)^71^. At 30 ± 2 °C, bacterial strains were grown for 24 h in LB broth with continuous stirring at 180 rpm to obtain the desired CFU of bacteria i.e. 1 × 10^8^ following centrifugation at 4 °C for 15 min for pelleting. Pellets were re-suspended very gently in 20 mL of chilled glycerol (10% v/v). The pelleting and re-suspending process was repeated and the volume of 10% (v/v) glycerol was reduced with a gradual decrease of 5 mL at each wash. Plasmid DNA was extracted from DH5α strain of *E. coli* containing pBBRIMCS-4 vector. It was an ampicillin-resistant 4.95 kb vector along with the YFP cassette^72^. QIAGEN QIA Mini-prep kit was used for plasmid isolation from overnight grown culture according to the manufacturer's standard protocol. Plasmid concentration was checked by Ultra spec3100 at OD 260 and 260/280. Electroporation was performed on Gene Probe (200 Ω resistor, 12.5 kV cm^−1^, 25 µF capacitor) with electro-competent cells and isolated plasmid DNA^71^. The selection of the transformed colonies was done by spreading 100 µL of this mixture on LB ampicillin agar plates that contains 50 µg mL^−1^ of ampicillin. The grown colonies were further confirmed by observing the cells of bacteria under CLSM on the glass slide *yfp* filter. These transformed cells were preserved at − 80 °C in glycerol (20% v/v).

### Root colonization studies using CLSM

The transformed bacterial isolates were grown at 28 ± 2 °C in 100 mL of ampicillin (50 μg mL^−1^) containing LB for 24 h up to 1 × 10^8^ CFU which were harvested at 8,000×*g* by centrifugation and re-suspended in 0.89% (w/v) NaCl. Medium size potato tubers were surface sterilized and inoculated with *yfp*-transformed strains. Inoculated tubers of potato were grown for 30 days in sterilized sand culture. Roots were cut, washed with a sterilized blade in to ½ cm pieces and analyzed under CLSM (Olympus fluo-view Ver. 1.3), on a glass slide with sterilized H_2_O covered with coverslip, to detect the colonization of *yfp*-labeled bacterial strains^13,73^.

### Plant inoculation assay

The bacterial strain was grown in 250 mL Erlenmeyer flasks containing 100 mL of LB broth and was constantly stirred at 180 rpm at 28 ± 2 °C up to 10^9^ CFU mL^−1^. Centrifugation at 8,000×*g* was done for harvesting of bacterial cultures and washed by 0.89% saline solution. After washing, the solution was again centrifuged for pelleting and the pellet was re-suspended in an equal amount of 0.89% saline solution. For evaluation of growth-promoting activity of isolates, a pot experiment under control conditions was conducted: 25 °C temperature and light and dark period of 16/8 with 400 mol m^−2^ S^−1^ photon flux density in completely randomized design (CRD) with 3 replicates of each treatment in sterilized sand. Sand was soaked for 24 h in 0.5 N Nitric acid for sterilization then washed with dH_2_O to remove acid which was further air-dried and autoclaved. Medium-sized potato (variety Kuroda) tubers (2–3 cm) were surface sterilized with sodium hypochlorite (8% v/v) for 10 min followed by extensive washing with sterilized dH_2_O. Sterilized potato tubers were immersed in bacterial inoculum for 30 min, dried for 10 min in Laminar Air flow cabinet and then sown in pots containing sterilized sand. The 2nd dose of bacterial inoculum was applied to potato roots after 7 days of sowing. The bacterial inoculum was mixed in Hoagland solution (without nitrogen source) and applied with sterilized syringe @ 5 mL/plant. There were 3 treatments with 3 replicates each, T1: un-inoculated control treatments; positive control (recommended full dose of N^F^), T2: negative control; without nitrogen (N^0^) and T3: inoculated treatment (Inoculated with bacterial isolate TN37 + N^0^ = without nitrogen). Growth parameters like shoot and root length, shoot and root fresh weight and dry weight and N contents of plants was recorded after 60 days of sowing.

### Statistical analysis

Analysis of variance (ANOVA) for different plant parameters was analyzed using STATISTIX8.1 software (Tallahassee, FL, USA)^74^. For comparing means of each treatment, the least significant difference test (LSD) was performed with 5% probability.

## Supplementary information

Supplementary Table S1.
