# Supplementary material for: First report of diazotrophic Brevundimonas spp. as growth enhancer and root colonizer of potato
Source: Sci Rep. 2020 Jul 30;10:12893. doi: 10.1038/s41598-020-69782-6 (PMC7393102; doi:10.1038/s41598-020-69782-6)
Supplement: Supplementary file 1 — Supplementary Table S1. [file 41598_2020_69782_MOESM1_ESM.pdf]

First Report of diazotrophic *Brevundimonas* spp. as growth enhancer and root colonizer of potato

Tahir Naqqash[1,2*], Asma Imran[2], Sohail Hameed[2,3], Muhammad Shahid[4], Afshan Majeed[2,5], Javed Iqbal[2], Muhammad Kashif Hanif[2,6], Shaghef Ejaz[7], Kauser Abdullah Malik[8]

Supplementary Table 1 Metabolic potential of selected bacterial isolate TN37 isolated from potato roots

Carbon Source	TN37	Carbon Source	TN37	Carbon Source	TN37
Chondroitin Sulfate C	-	α-Methyl-D-Mannoside	-	Quinic Acid	-
α-Cyclodextrin	-	β-Methyl-D-Xyloside	-	D-Ribono-1,4-Lactone	-
β-Cyclodextrin	-	Palatinose	-	Sebacic Acid	-
γ-Cyclodextrin	-	D-Raffinose	-	Sorbic Acid	-
Dextrin	-	Salicin	-	Succinamic Acid	-
Gelatin	-	Sedoheptulosan	-	D-Tartaric Acid	-
Glycogen	+	L-Sorbose	-	L-Tartaric Acid	-
Inulin	-	Stachyose	-	L-Alaninamide	-
Laminarin	-	D-Tagatose	-	N-Acetyl-L-Glutamic Acid	+
Mannan	+	Turanose	-	L-Arginine	-
Pectin	+	Xylitol	-	Glycine	-
N-Acetyl-D-Galactosamine	-	N-Acetyl-D-Glucosaminitol	-	L-Histidine	-
N-Acetyl-Neuraminic Acid	-	γ-Amino Butyric Acid	-	L-Homoserine	-
β-D-Allose	-	δ-Amino Valeric Acid	-	Hydroxy-L-Proline	-
Amygdalin	-	Butyric Acid	-	L-Isoleucine	-
D-Arabinose	+	Capric Acid	-	L-Leucine	-
D-Arabitol	-	Caproic Acid	-	L-Lysine	-
L-Arabitol	-	Citraconic Acid	-	L-Methionine	-
Arbutin	-	Citramalic Acid	-	L-Ornithine	-
2-Deoxy-D-Ribose	-	D-Glucosamine	+	L-Phenylalanine	-
i-Erythritol	-	2-Hydroxy Benzoic Acid	-	L-Pyroglutamic Acid	-
D-Fucose	+	4-Hydroxy Benzoic Acid	-	L-Valine	-

**3-0-β-D-Galacto-pyranosyl-D-Arabinose**	**-**	**β-Hydroxy Butyric Acid**	**-**	**D,L-Carnitine**	**-**
**Gentiobiose**	**-**	**γ-Hydroxy Butyric Acid**	**-**	**Sec-Butylamine**	**-**
**L-Glucose**	**-**	**α-Keto-Valeric Acid**	**-**	**D.L-Octopamine**	**-**
**Lactitol**	**-**	**Itaconic Acid**	**-**	**Putrescine**	**-**
**D-Melezitose**	**-**	**5-Keto-D-Gluconic Acid**	+	**Dihydroxy Acetone**	**-**
**Maltitol**	**-**	**D-Lactic Acid Methyl Ester**	**-**	**2,3-Butanediol**	**-**
**α-Methyl-D-Glucoside**	**-**	**Malonic Acid**	**-**	**2,3-Butanone**	**-**
**β-Methyl-D-Galactoside**	**-**	**Melibionic Acid**	**-**	**3-Hydroxy 2-Butanone**	**-**
**3-Methyl Glucose**	**-**	**Oxalic Acid**	**-**	**Acetamide**	**-**
**β-Methyl-D-Glucuronic Acid**	**-**	**Oxalomalic Acid**	+		
